# Indirubin as a red hair Colourant from *Indigofera tinctoria* L.

**DOI:** 10.1111/ics.13076

**Published:** 2025-05-21

**Authors:** Skrollan Klaas, Volkmar Vill, Fabian Straske

**Affiliations:** ^1^ Henkel AG & Co. KGaA Hamburg Germany; ^2^ Department of Chemistry University of Hamburg Hamburg Germany

**Keywords:** biosynthesis pathway, chemical analysis, colour cosmetics, hair treatment, indigo, spectroscopy

## Abstract

**Objective:**

Detailed understanding of the indigo and indirubin synthesis pathway to control their formation and the corresponding colour result on hair should be obtained. Managing the formation of the dye molecules indigo and indirubin the characteristic colour shift which takes place within the days after application of *Indigofera* *tinctoria L.‐based* hair colouration should be eliminated. Thus, intense, more reliable and stable colour results on hair from the natural resource *I. tinctoria* L. are achieved offering benefits to the consumer resulting in higher acceptance of plant‐based colouration products.

**Methods:**

Various colourations with *I. tinctoria* L. crushed leaves powder and cysteine, isatin or ascorbic acid on yak hair strands were carried out at room temperature or 37°C. Colour assessment was conducted with L*‐, a*‐, and b*‐values/coordinates of three‐dimensional CIE‐Lab‐colour. Coloured hair strands were pulverized with a ball mill to extract dyeing compounds from hair fibres with either a mixture of ultrapure water and acetonitrile or dimethyl sulfoxide at 80°C. Afterwards, the supernatant was collected, and dyeing compounds were quantified via high‐performance liquid chromatography.

**Results:**

It was proven that delayed development of indirubin in addition to the directly formed indigo molecule leads to the observed colour shift on hair fibres when dyeing them with *I. tinctoria* L. Further, a previously unseen, immediate formation of a stable red colour result was achieved by introducing isatin and cysteine or ascorbic acid to the dyeing procedure. By extraction and subsequent analysis of the dye molecules from the coloured hair fibres, it was confirmed that the addition of isatin and a reducing agent to indigo dyeing prevents the formation of the indigo molecule and favours the formation of indirubin.

**Conclusion:**

Based on the findings, including the instant formation of indirubin instead of the indigo molecule in the presence of isatin and cysteine or ascorbic acid causing an immediate stable red colour result after the application of *I. tinctoria* L. on hair, a revised indigo and indirubin synthesis pathway for *I. tinctoria* L. application including the effect of said additives to the colour outcome is presented.

## Graphical abstract

An immediate stable red colour result after application of *Indigofera tinctoria* L. on hair in the presence of isatin and a reducing agent was achieved. A revised indigo molecule and indirubin synthesis pathway for *I. tinctoria* L. application including the effect of said additives to the colour outcome is presented.

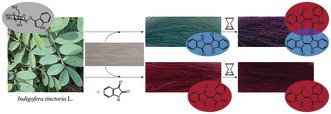

## Résumé

**Objectif:**

Une compréhension détaillée de la voie de synthèse de l'indigo et de l'indirubine devrait être obtenue afin de contrôler leur formation et le résultat de la couleur correspondante sur les cheveux. La gestion de la formation des molécules colorantes indigo et indirubine devrait permettre d'éliminer le changement de couleur caractéristique qui se produit dans les jours suivant l'application d'une coloration capillaire à base d'*Indigofera tinctoria* L. Ainsi, des résultats de coloration intenses, plus fiables et plus stables sur les cheveux à partir de la ressource naturelle *Indigofera tinctoria* L. sont obtenus, ce qui présente des avantages pour le consommateur et entraîne une plus grande acceptation des produits de coloration à base de plantes.

**Méthodes:**

Diverses colorations avec la poudre de feuilles broyées d'*Indigofera tinctoria* L. et la cystéine, l'isatine ou l'acide ascorbique sur des mèches de cheveux de yak ont été réalisées à température ambiante ou à 37°C. L'évaluation de la couleur a été effectuée à l'aide des valeurs/coordonnées L*‐, a*‐ et b* de la couleur tridimensionnelle CIE‐Lab. Les mèches de cheveux colorés ont été pulvérisées à l'aide d'un broyeur à billes pour extraire les composés colorants des fibres capillaires avec un mélange d'eau ultrapure et d'acétonitrile ou de sulfoxide de diméthyle à 80°C. Le surnageant a ensuite été éliminé à l'aide d'une solution aqueuse. Le surnageant a ensuite été recueilli et les composés colorants ont été quantifiés par chromatographie liquide haute performance.

**Résultats:**

Il a été prouvé que le développement retardé de l'indirubine en plus de la molécule d'indigo directement formée conduit au changement de couleur observé sur les fibres capillaires lors de la teinture avec *Indigofera tinctoria* L. En outre, une formation immédiate d'une couleur rouge stable, jamais observée auparavant, a été obtenue en introduisant de l'isatine et de la cystéine ou de l'acide ascorbique dans la procédure de teinture. L'extraction et l'analyse ultérieure des molécules de colorant des fibres capillaires colorées ont confirmé que l'ajout d'isatine et d'un agent réducteur à la teinture à l'indigo empêche la formation de la molécule d'indigo et favorise la formation de l'indirubine.

**Conclusion:**

Sur la base des résultats, y compris la formation instantanée d'indirubine au lieu de la molécule d'indigo en présence d'isatine et de cystéine ou d'acide ascorbique provoquant une couleur rouge stable immédiate après l'application d'*Indigofera tinctoria* L. sur les cheveux, une voie de synthèse révisée de l'indigo et de l'indirubine pour l'application d'*Indigofera tinctoria* L., y compris l'effet de ces additifs sur le résultat de la couleur, est présentée.

## INTRODUCTION

The indigo molecule has been one of the best‐known dyeing components in the textile industry for more than 2000 years. The indigo plant (*I. tinctoria* L.) used for dyeing is mainly grown in India. During the 18th century, the production of synthetic indigo arose and the demand for naturally derived indigo declined since it was then possible to produce it in a purer and more affordable way [1, 2]. In contrast to natural indigo, both the process for synthetic indigo production and colouration are highly energy demanding and hazardous for the environment due to large amounts of toxic waste. With increasing awareness of the environmental impact, the demand for plant‐based indigo colouration is on the rise again [3, 4].

Besides its use in the textile industry, *I. tinctoria* L. is used as a traditional hair dye where the dried and shredded leaves are mixed with water and applied on hair. As water is added to the leaves of *I. tinctoria* L. for hair colouration purposes, the indigo biopathway is initiated. The intact cell structure of the plant's leaves is destroyed through the shredding; therefore, precursor and enzyme are able to come into contact. The addition of water brings the last missing component for triggering the dyeing procedure [5]. Its colouration appears blue freshly after application. The specialty of this colouration is the colour shift. The blue coloured hair turns purple in the days following the application. However, this is an issue as consumers want a stable colour result immediately after application. Therefore, in a market launch of a competitive hair colouration product, it is essential that the colour result gets stabilized, and the colour formation process is controlled.

To understand and control extraction and subsequent hair colouration with *I. tinctoria* L. it is crucial to consider the corresponding biosynthesis pathways of dye formation. In the plant's leaves vacuoles, the dye is stored in form of the colourless stable precursor indican (indoxyl‐β‐D‐glucoside) [5, 6]. The plant's own β‐glucosidase is stored in chloroplasts. As soon as the leaves lose their intact structure and are exposed to water and oxygen, the hydrolytic deglycosylation of indican caused by the plant's own enzyme is initiated and indoxyl is formed [5, 7, 8, 9]. This is followed by a dimerization of indoxyl by air oxygen to the formation of indigo molecule (2‐(1,3‐Dihydro‐3‐oxo‐2H‐indol‐2‐ylidene)‐1,2‐dihydro‐3H‐indol‐3‐one) [10, 11]. In addition, as a side‐reaction in an oxygen‐rich environment, isatin is formed from indoxyl. Through condensation, isatin and indoxyl together form the red molecule indirubin (3‐(1,3‐Dihydro‐3‐oxo‐2H‐indol‐2‐yliden)‐1,3‐dihydro‐2H‐indol‐2‐one) [12]. Indirubin as a structural isomer of indigo molecule absorbs shorter wavelengths than indigo and therefore appears in a red shade. While both indigo and indirubin command extensive intermolecular hydrogen bonding in its crystal structure, indirubin shows more restricted intramolecular hydrogen bonding [12, 13].

To manipulate the dye formation from *I. tinctoria* L. raw material, reducing agents like cysteine can be used. The major product shifts from the indigo molecule to indirubin upon the addition of cysteine [14]. To explain the points of action of cysteine in the biosynthesis pathway, Kim et al. (2019) firstly introduce the prevention of oxidized indoxyl from forming the indigo molecule by developing an intermediate product called 2‐cysteinylindoleninone in a non‐enzymatic, oxidative way. This intermediate product reacts with isatin spontaneously to indirubin unless the availability of isatin as a reactant is limited. The isatin synthesis might take place by the hydrolysis of 2‐cysteinylindoleninone or the oxidation of indoxyl at C2. Beyond that, Kim et al. (2019) assume that cysteine promotes the reaction of isatin with 2‐cysteinylindoleninone resulting in indirubin formation [15].

In this research, a new approach to control the colour result with *I. tinctoria* L. is presented and adjustments to the existing biosynthesis pathway are proposed.

## MATERIALS AND METHODS

### Hair colouration protocol

Untreated, white, selected yak hair strands from Kerling Haarfabrik GmbH (Backnang, Germany) were used for all experiments. The total length of a strand incl. plastic bonding was 11.8 cm and the length of free hair fibres amounted to approx. 7.5 cm with a weight of 0.7 g. Before colouration treatments, the strands were cleaned based on a standardized protocol. First, the strands were moistened while combing with a tap water flow of 25 mL s^−1^. Next, they were placed in 20 mL/strand of an aqueous 12.5% sodium laureth sulfate (SLES) solution adjusted to pH 4.5 (HCl, 10%) for 30 min at room temperature (RT). The strands were then rinsed with tap water at a flow of 25 mL s^−1^ and combed approx. 10 times until no further SLES bubbles were visible. Finally, the strands were air‐dried overnight and ready for further treatment.

Dyeing experiments were carried out with *I. tinctoria* L. crushed leaves powder (InCL) supplied by Kremer Pigmente GmbH & Co. KG. The plant is cultivated yearly between December and April, and there are three harvests in a single cultivation. The first harvest period is 3 months from the time of cultivation, the second harvest occurs 50 days after the first and the third harvest another 50 days later. The region of the cultivation area is Viluppuram, India [16].

For further dyeing experiments, (R)‐(+)‐cysteine (for synthesis, CAS: 52–90‐4) was purchased from Merck KGaA (Darmstadt, Germany) and isatin (98%, CAS: 91–56‐5) from ThermoFisher GmbH (Kandel, Germany). Hydrochloric acid (HCl 37%, CAS: 7647‐01‐0) was purchased from VWR International GmbH (Darmstadt, Germany). HCl aqueous dilution to 10% was freshly prepared in the laboratory.

Colouration was carried out by adding plant powder into 50 mL of de‐ionized water and stirring it with the strand for 30 min following the protocol of Sargsyan et al. [17]. For InCL colouration, 5% (w/v) of plant material was suspended in 50 mL of de‐ionized water with a natural pH of 6.8.

Moreover, 1% (w/v) cysteine was added to the dyeing suspension with the same natural pH. Another colouring approach was the addition of 1% (w/v) isatin and cysteine to InCL suspension at RT or at 37°C with a natural pH of 6.1 or 5.8, respectively. The addition of each 1% (w/v) isatin and ascorbic acid lead to a natural pH of InCL suspension of 2.7 at 37°C.

After dyeing, the strands were rinsed with tap water at RT with a total tap water flow of 25 mL s^−1^ and combed until the dyeing mixture had been removed. Subsequently, the strands were blow dried with a commercially available blow‐dryer at approx. 80°C. Strands were maintained at RT, without shielding from UV radiation and without any intermediate washing between day 0 and day 14 after colouration treatment.

### Colour assessment

Colour assessment was conducted with L*‐, a*‐, and b*‐values/coordinates of the three‐dimensional CIE‐Lab‐colour spectrum by Spectraflash 600 X (Cataloguer.: 1200–1405, Serialnr.: 8633) of Datacolor, Applied Color Systems, Inc.

Colourimetric data were recorded in DCI colour software. Each strand was measured four times with a turn of the strand at 90° in between every value recording. The mean value of the four measurements was used for colour assessment and further calculations.

Difference in colours between strands was assessed by ΔE value, calculated as follows [18]:

(1) $$∆E=\sqrt{\left({\left(∆L^{*}\right)}^{2}+{\left(∆a^{*}\right)}^{2}+{\left(∆b^{*}\right)}^{2}\right)}$$

### HPLC analysis

Ultrapure Water (UPW) was used for all HPLC experiments. The purification was conducted using the Arium Pro Ultrapure Water System (Sartorius AG). Acetonitrile (CH_3_CN; ACN) purchased from CARLO ERBA Reagents GmbH (CAS: 75–05‐8; Emmendingen, Germany) and dimethyl sulfoxide (C_2_H_6_OS; DMSO) purchased from ThermoFisher GmbH (CAS: 67–68‐5) were of HPLC grade.

For preparation of calibration curves, isatin (98%, CAS: 91–56‐5, ThermoFisher GmbH) was dissolved in ACN and UPW (45:55). Indigo (94%, synthetic, CAS: 482–89‐3, C.I.73000, Acros Organics) and indirubin (≥98%, CAS: 479–41‐4, Cayman Chemicals) were diluted in DMSO.

Dilutions of 20, 15, 10, 7,5, 6, 5, 3, 2,5, 1,5 1 and 0,5 mg L^−1^ were analysed using Knauer AZURA® Analytical HPLC with Autosampler AS 6.1 L (Knauer GmbH) and an analytical column (Eurospher II 100–5 C18, 250 × 4.6 mm, 5 μm, 100 Å, Knauer GmbH) attached to HPLC at 40°C (column thermostat AZURA® CT 2.1, Knauer GmbH).

ACN (solvent A) and UPW with 0.1% formic acid (solvent B) were gradually used (flow rate 0.6 mL min^−1^) as follows: 0^−1^ min 5% A, 1–20 min 40% A, 20–25 min 100% A, 25–32 min 100% A, 32–35 min 100% A and 35–40 min 5% A.

Compounds were detected by AZURA Detector DAD 2.1 L (Knauer GmbH). Collected data were analysed with ClarityChrom®9 (Knauer GmbH).

### Processing of hair strands for analytics

For investigation on hair colouration with InCL analogously to Mantzouris et al., differently coloured strands were stirred in 50 mL UPW strand^−1^ for 60 min, whereby the water was changed twice in total. Further, dried strands were ground with the help of a Pulverisette 23 ball mill (Fritsch GmbH) which was equipped with a zirconium oxide grinding bowl and balls. The runtime was 2 × 5 min with a cool‐down time of 2–3 min in between and a frequency of 50 s^−1^ [19].

Afterwards, 8 mg of powdered strand was mixed with 2 mL of either UPW/ACN or DMSO in a tube and subsequently inserted into a thermal shaker (ThermoMixer® C) at 80°C and 1000 rpm for 30 min. The samples were then transferred into a centrifuge (Centrifuge 5910 Ri) with Rotor S‐4xUniversal for 20 min at RT and 4347 × g [20]. In accordance with [19] the supernatant was used for HPLC analysis. The chromatographic peaks were identified and quantified by the retention time and integrals of standards. Furthermore, the results were converted to μg molecule g strand^−1^ for better comparability. Each experiment for molecule determination with HPLC was carried out five times.

### Statistical analysis

Statistical analysis of experiments was carried out with IBM SPSS Statistics Version 29.0 (IBM Corp.). Normal distribution of the data was assumed. Results are presented as mean values ± standard deviation (SD).

## RESULTS AND DISCUSSION

### Hair colouration with *I. tinctoria* L.

**FIGURE 1:**
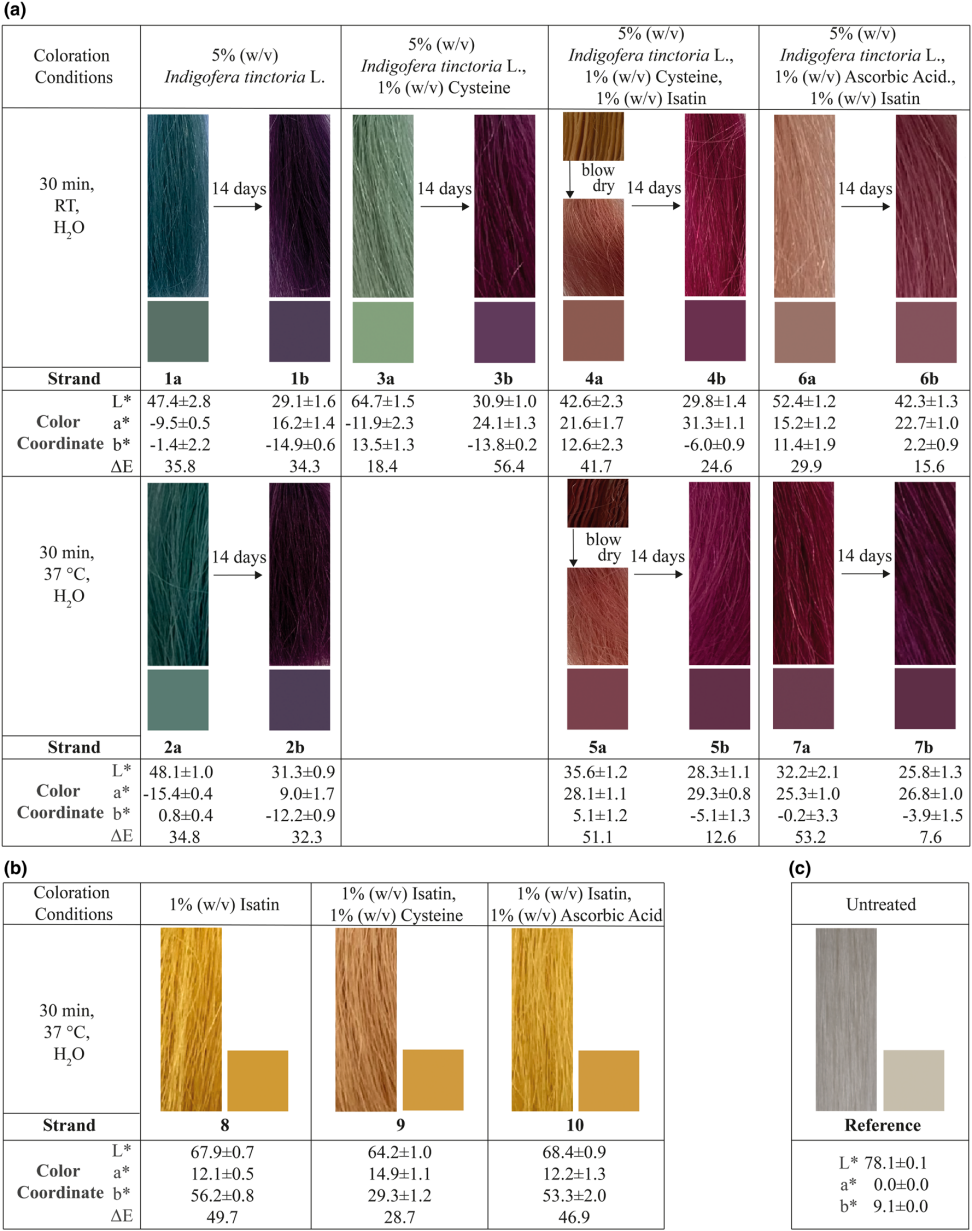
Different colourations on yak hair with CIE‐Lab values; (a) Colourations with 5% (w/v) *Indigofera tinctoria* L. freshly coloured and on day 14 at RT (1a,b), freshly coloured and on day 14 at 37°C (2a,b), with the addition of 1% (w/v) cysteine freshly coloured and on day 14 at RT (3a,b), with the addition of 1% (w/v) cysteine and 1% (w/v) isatin freshly coloured and on day 14 at RT (4a,b), freshly coloured and on day 14 at 37°C (5a,b), with the addition of 1% (w/v) ascorbic acid and 1% (w/v) isatin freshly coloured and on day 14 at RT (6a,b), freshly coloured and on day 14 at 37°C (7a,b). (b) Control colourations without *I. tinctoria* L. with 1% (w/v) isatin freshly coloured at RT (8), 1% (w/v) isatin with 1% (w/v) cysteine freshly coloured at RT (9) and 1% (w/v) isatin with 1% (w/v) ascorbic acid freshly coloured at RT (10). Strands were maintained at RT, without shielding from UV radiation and without any intermediate washing between day 0 and day 14. (c) Untreated yak hair as a reference strand.

Hair colouration with *I. tinctoria* L. crushed leaves powder (InCL) leads to a shift in colour by ΔE = 34.3 from initial blue to purple after 14 days without special treatment (Figure 1 and Table 1, strands 1a + b, 2a + b). Strands were maintained at ambient temperature, without shielding from UV radiation and without any intermediate washing between day 0 and day 14. Understanding colour formation in the indigo biosynthesis process [5, 12, 14, 15] suggests that, alongside the indigo molecule, indirubin forms over time through oxygen‐driven side reactions using oxygen as an oxidizing agent. The overlap of both molecules may result in the perception of a purple hue [21, 22]. To verify cysteine's influence on the colouration process on hair, InCL was mixed with 1% (w/v) cysteine (colourless) in suspension and coloured on yak hair at RT (Figure 1 and Table 1, strands 3a + b). The natural pH of the cysteine and InCL dispersion was 6.9. On day 0 after colouration, a green hue can be detected on the hair fibre. Within 14 days the colour changes towards a darker, more red and blue shade with a measured difference of ΔE = 56.5 in regard to the colouration on day 0. In comparison, the strands coloured with InCL without the addition of cysteine differ from the one with cysteine on day 0 by ΔE = 23.0 and on day 14 by ΔE = 8.2 (Figure 1 and Table 1, strands 1a + b, 3a + b).

**TABLE 1:** Concentration of indigo molecule, indirubin, and isatin on strands coloured with 5% (w/v) *I. tinctoria* L. in water at 37°C freshly after dyeing procedure (2a) and after 14 days (2b) and strands coloured with 5% *I. tinctoria* L., 1% (w/v) Ascorbic Acid, and 1% (w/v) Isatin at 37°C freshly after dyeing procedure (7a) and after 14 days (7b).

Strand	Molecules (mg g strand^−1^)		
	Indigo	Indirubin	Isatin
2a	189.5 ± 40	0.0 ± 0.0	574.2 ± 70
7a	10.5 ± 0.0	467.7 ± 60	2378.1 ± 30
2b	137.5 ± 30	271.4 ± 50	237.5 ± 140
7b	2.1 ± 0.0	1866.9 ± 190	550.7 ± 320

This means the influence of cysteine on indigo colour outcome, and therefore biosynthesis pathway within hair colouration application, is indisputable, as the indigo molecule formation is hindered when freshly dyed due to the addition of cysteine to colour suspension. Furthermore, red colour formation after 14 days is promoted. Merely indoxyl molecules are responsible for the formation of the indigo molecule. However, instead of two indoxyl molecules, one indoxyl and one isatin molecule which is formed during the indigo synthesis pathway as a by‐product, react and form the red indirubin molecule in a condensation reaction [8, 15, 22].

To investigate isatin as a time‐determining factor in indirubin synthesis, cysteine and isatin were added for colouration. During rinsing of the colour suspension, it was observed that a slight rose‐coloured shade appeared on the strands. Only when the strands were blow dried (at ~80°C), the red shade appear (Figure 1, strands 4a + b, before and after blow drying).

Since the temperature applied during blow drying influenced the colour development on the strand, the same staining was carried out applying the suspension at 37°C instead of room temperature (RT) (Figure 1 and Table 1, strands 5a + b). The intensification of colour outcome with InCL, cysteine and isatin suspension at 37°C compared to RT amounts to ΔE = 12.1 on day 0. However, after 14 days, the strands are equalized and show a difference of ΔE = 2.7 between strands dyed with InCL, cysteine and isatin at RT and at 37°C. Further, with ΔE = 12.6, the colour of strands treated at 37°C changes within 14 days less than those coloured at RT (ΔE = 24.6). The temperature of 37°C is known from literature for incubation of β‐glucosidases [23, 24]. Therefore, the indoxyl might be released faster than at RT. Moreover, higher temperatures lead to higher diffusion rates and therefore better colour outcome after dyeing procedure.

Higher temperatures may enhance indoxyl and isatin conversion kinetics to indirubin, causing the red colour occurring immediately after treatment. However, there is an optimal temperature for the reaction, and the enzyme catalysing the hydrolysis of indican may denature if the temperature is exceeded [25].

By adding isatin and cysteine to InCL suspension and colouration at 37°C, an immediate red colour result is achieved. To the best of our knowledge, this is a novelty use of InCL for hair colouring. Further, the colour change between day 0 and day 14 differs less than in strands only coloured with InCL. If ascorbic acid in combination with isatin is used instead of cysteine, the hair colouration results are even improved in terms of intensity and stability. In contrast to Kim et al.'s observations in microbial biosynthesis, no specificity in the reducing agent can be recognized in relation to hair dyeing with *I. tinctoria* L. [15]. The colour of the strands treated with ascorbic acid and isatin at 37°C and the ones with cysteine and isatin at 37°C differ by ΔE = 6.9 on day 0. The strands dyed in the presence of ascorbic acid appear more intense and darker. After 14 days, the difference between these strands decreases to ΔE = 3.7. The strands treated with ascorbic acid and isatin at 37°C only differ by ΔE = 7.6 within 14 days, whereas the cysteine with isatin ones differ by ΔE = 12.6 (Figure 1 and Table 1, strands 5a + b, 7a + b).

The initial staining results are improved with ascorbic acid, and the stability of the colour within 14 days is also higher compared to the colouration with only cysteine at RT (ΔE = 56.4) (Table 1, strands 3a + b, 6a + b). This excludes the direct transferability of Kim et al.'s and Han et al.'s theory to our application with the plant material used as a hair dye. In case no cysteine is added, it is assumed that no intermediate product 2‐cysteinylindoleninone is formed and suggests that the reduction of the intermediate products is sufficient to prevent the major formation of indigo molecule on hair [14, 15].

As a control, in order to exclude the all‐encompassing influence of isatin, cysteine and ascorbic acid, strands were treated with different combinations of the additives without *I. tinctoria* L. (Figure 1, strands 8–10). These results prove the irreplaceability of InCL plant material for the red dyeing results. All in all, it is assumed that the addition of isatin and cysteine or ascorbic acid to InCL dyeing prevents the formation of indigo molecule and stimulates the formation of indirubin, resulting in a stable red colouration immediately after application.

### Chemical analysis of dye compounds on Coloured hair strands

**FIGURE 2:**
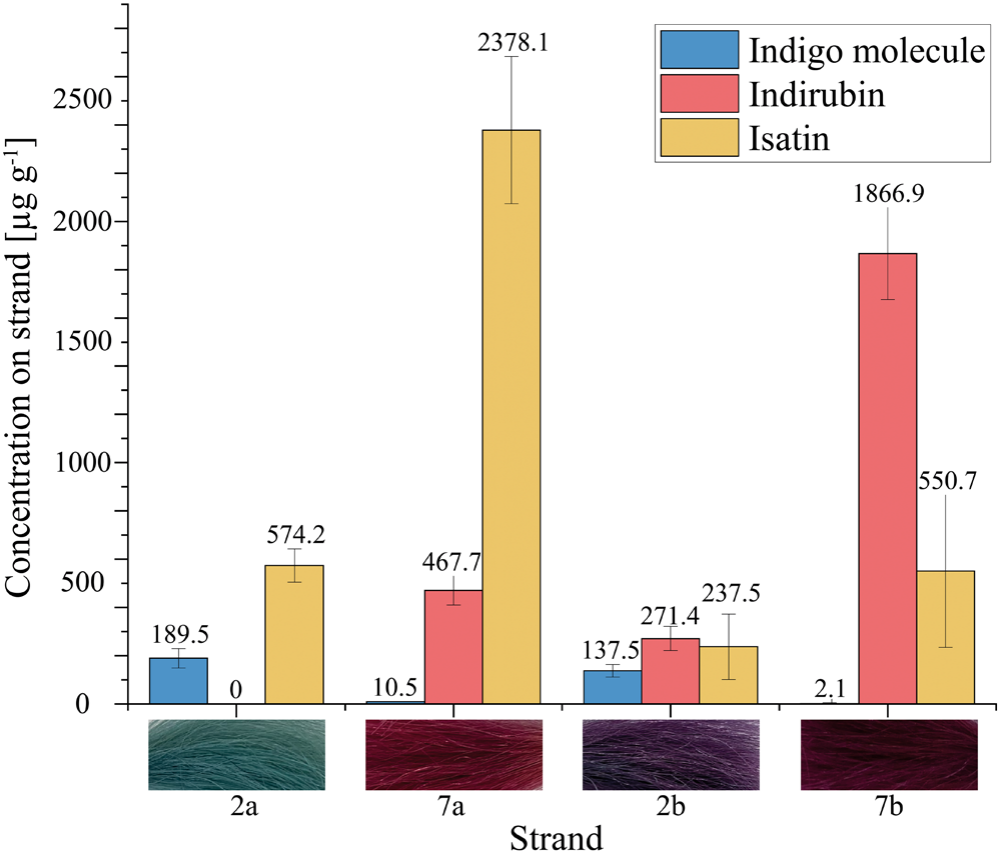
Bar chart of concentration of Indigo molecule, indirubin and isatin on strands coloured with 5% (w/v) *Indigofera tinctoria* L. in water at 37°C freshly after dyeing procedure (2a) and after 14 days (2b) and strands coloured with 5% *I. tinctoria* L., 1% (w/v) ascorbic acid and 1% (w/v) isatin at 37°C freshly after dyeing procedure (7a) and after 14 days (7b). Image of the corresponding‐coloured strands below.

The extraction and subsequent quantification of the colour molecules from hair fibres was carried out with strands from two different dye preparations—each fresh after dyeing and 14 days after application. The results for the strands treated with 5% (w/v) InCL in water freshly after dyeing procedure (2a) and after 14 days (2b) as well as 5% (w/v) InCL with 1% (w/v) ascorbic acid and 1% (w/v) isatin freshly after dyeing procedure (7a) and after 14 days (7b) prove the previously stated hypothesis to be true—the purple colour shift is due to the overlay of indigo and indirubin molecules (Figure 2). Both colouration approaches were performed at 37°C for best comparability and all strands were separately stirred in water for 60 min and blow dried again directly before examination. This was done to prevent the extraction of colour precursors that remain on the hair surface and were not bonded to the hair fibre.

The difference in colour of strands treated with 5% (w/v) InCL freshly coloured and 14 days after colouration is depictable in L*a*b*‐colour difference with ΔE = 32.3. Initially, the strands which appear blue right after the dyeing process yield 189.5 μg g strand^−1^ indigo molecule and no indirubin. Strands subjected to the same protocol that were washed and examined 14 days after colouration with 5% (w/v) InCL, appearing purple, only show 137.5 μg g strand^−1^ indigo molecule, a decrease of 27.4% compared to the strand at day 0. At the same time, the amount of detectable indirubin increases to 271.4 μg g strand^−1^. In summary, this leads to the overall purple colour impression on hair.

On the one hand, experiments by Tello‐Burgos et al. (2020) and Novotná et al. (2003) have shown that, due to photooxidation, indigo also decomposes over time in the solid state into isatin, among other degradation products [26, 27, 28]. It is inferred that the resulting isatin could be available for indirubin synthesis. This explains the decrease in the indigo molecule concentration on the hair and the delayed formation of indirubin within 14 days. Furthermore, it is assumed that the influence of stirring the strands on colour development is also related to indigo degradation. As colour precursors that do not bind to the hair are largely extracted, they are subsequently no longer available for indigo or indirubin synthesis. As soon as the indigo degrades into isatin, thereby making it available again on the fibre, the component to form indirubin is missing. This prevents the hair colour from changing to reddish. On the other hand, the decrease in indigo molecule detection on strands from freshly coloured to day 14 after colouration could be based on the experimental set up, since different strands were tested for their molecular content at both times.

The largest quantities of indigo and indirubin molecules in total were extracted from the red coloured strands with 5% (w/v) InCL, 1% (w/v) ascorbic acid and 1% (w/v) isatin at 37°C both on day 0 (7a) and day 14 (7b). Although compared to the freshly blue coloured strand, 94.5% less indigo molecule could be detected on strands 7a, the total amount of molecules detected exceeds 2a by 60.4%. This result continues to intensify within 14 days: the quantity of indigo molecules decreases further by 80%, and the quantity of indirubin increases by 74.9% when comparing strands 7a (day 0) and 7b (day 14). The difference between the red appearing strands regarding the L*a*b*‐colour measurement is reflected with ΔE of 7.6.

As assumed after dyeing experiments and L*a*b*‐measurements, it was possible to identify different amounts of colour molecules on the hair fibre, which are responsible for the visible colour impression. However, the higher intensity of the red colour on the strands 7a to 7b is reflected more prominently in the increasing amount of indirubin detectable on strand than in ΔE. Even if such high quantities of molecules are not detectable on the 2a and 2b strands, the L*a*b*‐colour impression is clearly distinguishable. The difference between these two examples is that the a*‐ and b*‐values of the red strands have remained stable over time (Δa: 1.6; Δb: 3.7) and only the L*‐value has changed by 6.4. In the colour development of 2a to 2b strands, there is a change in all three values of at least 13 (ΔL: 16.7; Δa: 24,4; Δb: 13). Overall, it can be concluded that the visible colour results on the hair are related to the mixture of indigo and indirubin molecules and their proportions detected rather than the total amounts of dye molecules.

Regarding the extraction of dye precursor isatin from hair fibre, further insights into the formation of hair colouration after application of InCL are provided. Freshly coloured strands with InCL exhibit 574.2 μg g strand^−1^ isatin. During purple colour development on the hair fibre (2b), the amount of isatin is reduced by 58.4% to 237.5 μg g strand^−1^. The same applies to the measured isatin of 2378.1 μg g strand^−1^ on the freshly coloured red strand which is reduced by 76,8% to 550.7 μg g strand^−1^ in the course of 14 days after application. The higher quantity of isatin on red coloured strands in the first place can be explained by the fact that the substance was initially added to the colouring suspension. In addition, no statement can be made about isatin amounts of indigo degradation, as the influence cannot be conclusively assessed by examining different strands within this experiment. Therefore, it is not the absolute values of isatin between the differently treated strands that are compared, but the ratios of the amount of substance on strands treated in the same way over the course of 14 days.

These decreases in the detectable quantity of isatin and indigo molecule, as well as the simultaneous increase in indirubin in both applications of InCL, indicate that isatin contributed as an educt for the indirubin synthesis. The degradation of the substance over time then speaks for its indispensability for indirubin synthesis. Further, these findings support the assumption that the slow formation of indirubin on hair treated with InCL is due to the lack of isatin in the system in the first place [8, 15].

### *I. tinctoria* L. synthesis pathway

**FIGURE 3:**
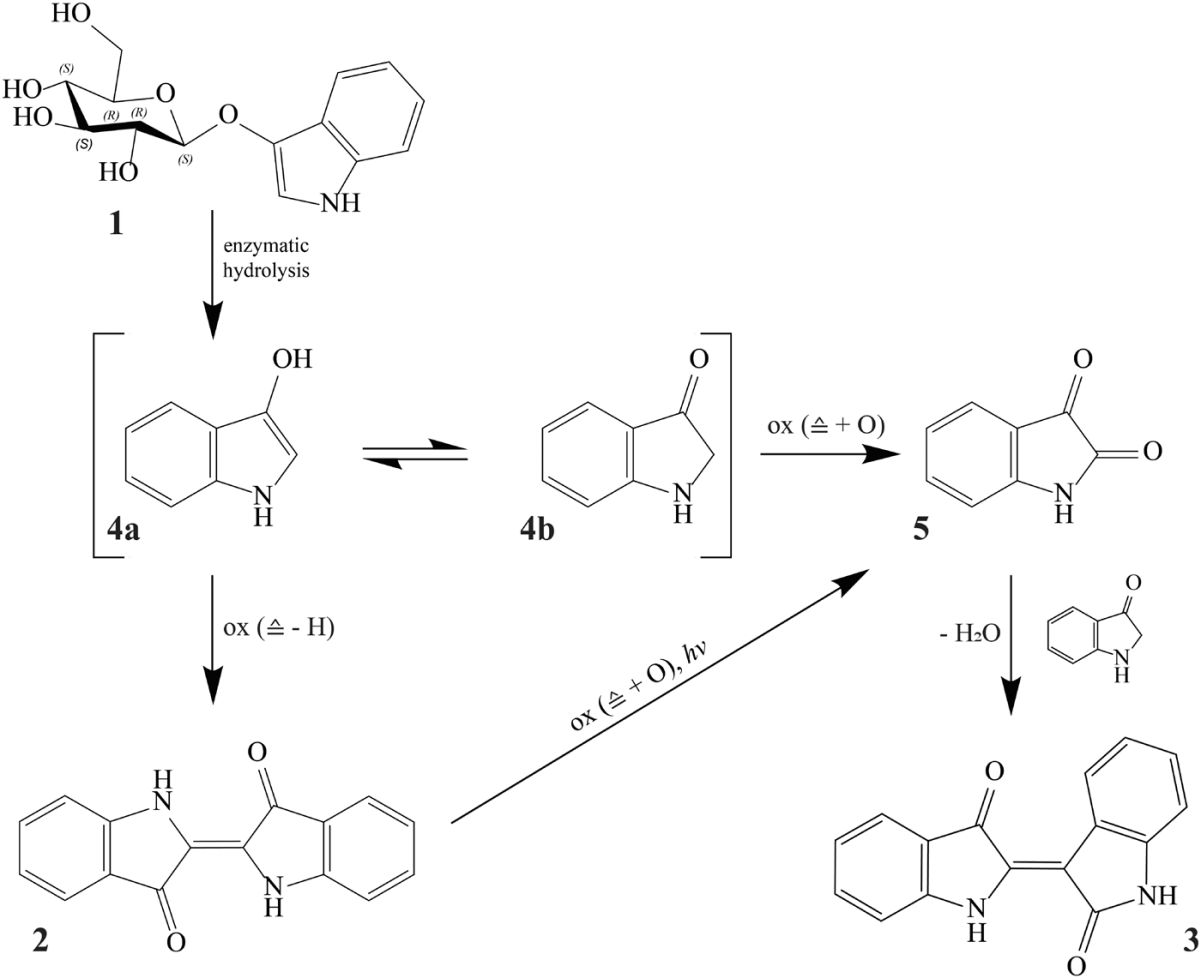
Proposed indigo molecule/indirubin synthesis pathway from *Indigofera tinctoria* L. in accordance with Blackburn et al. [22]; 1: indican, 2: indigo molecule, 3: indirubin, 4a, 4b: Indoxyl molecules, 5: Isatin. To maintain clarity, precise stoichiometric balances have been omitted, as the primary aim is to highlight the oxidative reaction pathways involved.

According to dye synthesis pathways described for *I. tinctoria* L. so far and the results of this research, a revised reaction scheme for hair colouration with *I. tinctoria* L. is proposed (Figure 3) [15, 22].

After deglycolization of the indican molecule (1) through enzymatic hydrolysis and development of indoxyl enol (4a) or keto (4b) tautomeric form, two different oxidation reactions can take place. The indigo molecule (2) results from the dimerization of two indoxyl molecules (oxidative coupling) or isatin (5) formation through spontaneous oxidation [5, 8, 15, 23, 29] Indigo molecule can also be degraded by photooxidation into isatin molecules [26, 27, 28]. In the next step, through condensation of indoxyl and isatin, indirubin (3) is formed. As already shown based on hair colouration, indirubin synthesis takes longer than the synthesis of the indigo molecule. The speed‐determining step in this pathway is the conversion of indoxyl into isatin, due to the different speeds of different oxidation steps. Therefore, indirubin synthesis can be sped up with the addition of external isatin into the system.

In addition, the pathway can be influenced by adding H_2_‐donors, such as ascorbic acid or cysteine. These substances prevent indigo molecule formation and thus cause an increase in indoxyl molecule concentration. Consequently, the dye precursors are available for isatin synthesis and thus indirubin synthesis to a greater extent [15]. Further promotion of the reaction can be achieved by increasing the reaction temperature to 37°C, which is in line with the previously proposed indigo and indirubin synthesis pathway. The pathway has not been investigated at the molecular level but can be deduced from the observations described.

## CONCLUSION

Based on the results of this research, it can be concluded that the typical colour change from initial blue to purple when colouring hair with *Indigofera tinctoria* L. crushed leaves was caused by the additional formation of indirubin on the hair fibre. The initial formation and attachment of indigo dye to the hair fibre resulted in the characteristic blue staining of the hair tresses. Over time, the red dye indirubin is formed and resulted in a purple hue due to the overlay of blue and red colourants indigo and indirubin. The premise that the addition of isatin and cysteine to indigo dyeing prevented the formation of the indigo molecule and stimulated the formation of indirubin was confirmed. Through the addition of a reducing agent, the formation of the indigo molecule was prevented. Further, with the addition of isatin to the staining suspension, the production of indirubin on hair was promoted, especially at 37°C reaction temperature. This proves that the lack of isatin was the limiting factor in the natural indirubin synthesis. Consequently, it was possible to achieve a red hair dyeing result directly after application with *Indigofera tinctoria* L. crushed leaves for the first time. A revised indigo and indirubin synthesis pathway from *Indigofera tinctoria* L. for application to the hair, including the effect of additives to influence colour outcome, was presented. The findings provided a basis for controlling the shade of hair colouration with indican from *Indigofera tinctoria* L. and therefore, addressed the needs of consumers.

## CONFLICT OF INTEREST STATEMENT

The authors declare that they have no conflicts of interest regarding the publication of this article.
